# Supplementary material for: B Lymphocyte Development in the Bursa of Fabricius of Young Broilers is Influenced by the Gut Microbiota
Source: Microbiol Spectr. 2023 Mar 14;11(2):e04799-22. doi: 10.1128/spectrum.04799-22 (PMC10100789; doi:10.1128/spectrum.04799-22)
Supplement: Supplemental file 1 — Supplemental material. Download spectrum.04799-22-s0001.pdf, PDF file, 1.9 MB [file spectrum.04799-22-s0001.pdf]

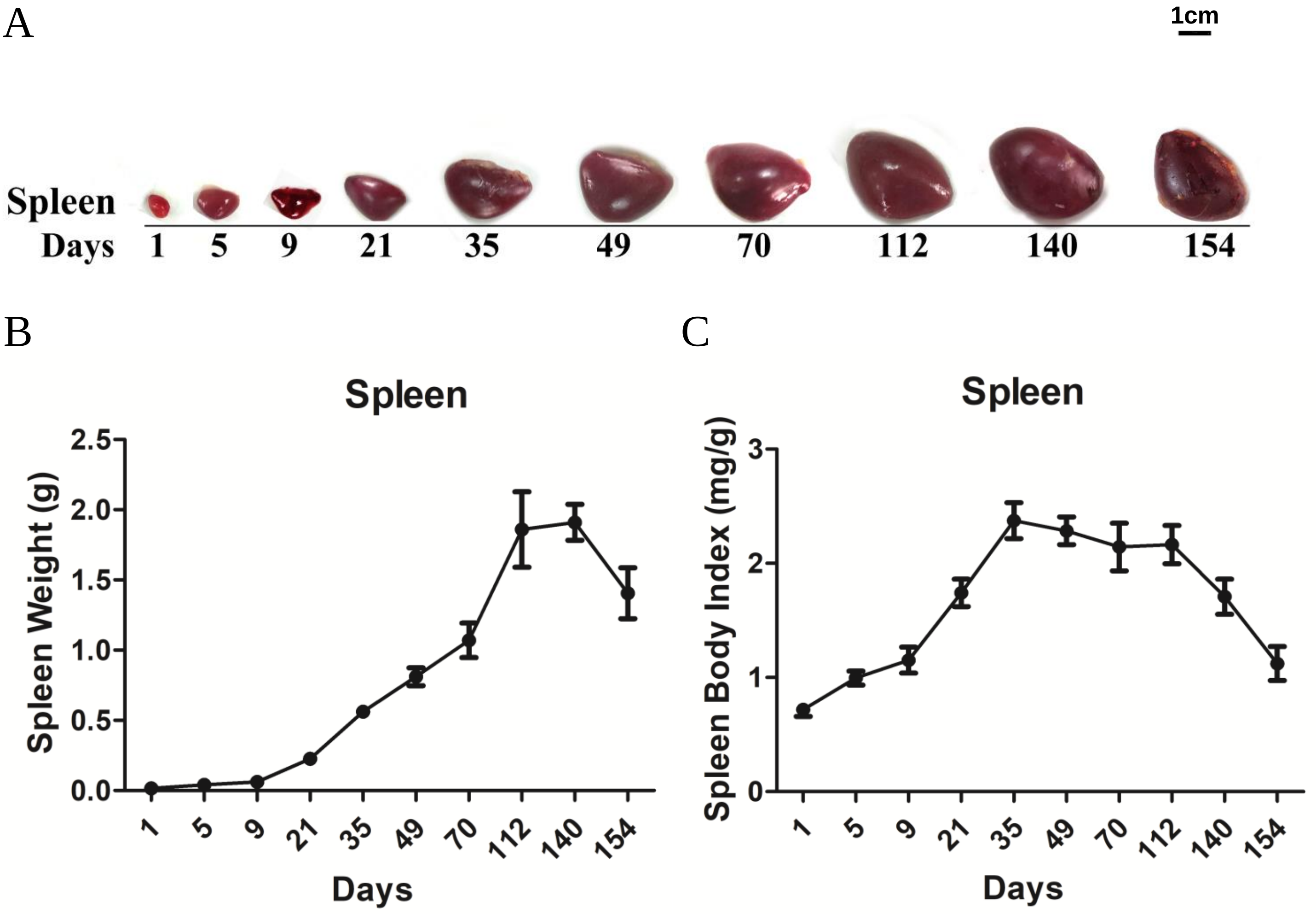

Figure S1. Size and weight of the spleen at different ages. (A) Size of spleen from 1 to 154 days. (B) Weight of spleen from 1 to 154 days. (C) Ratio of spleen to body weight from 1 to 154 days. Data are means ± SEM (*$P < 0.05$), n = 6.

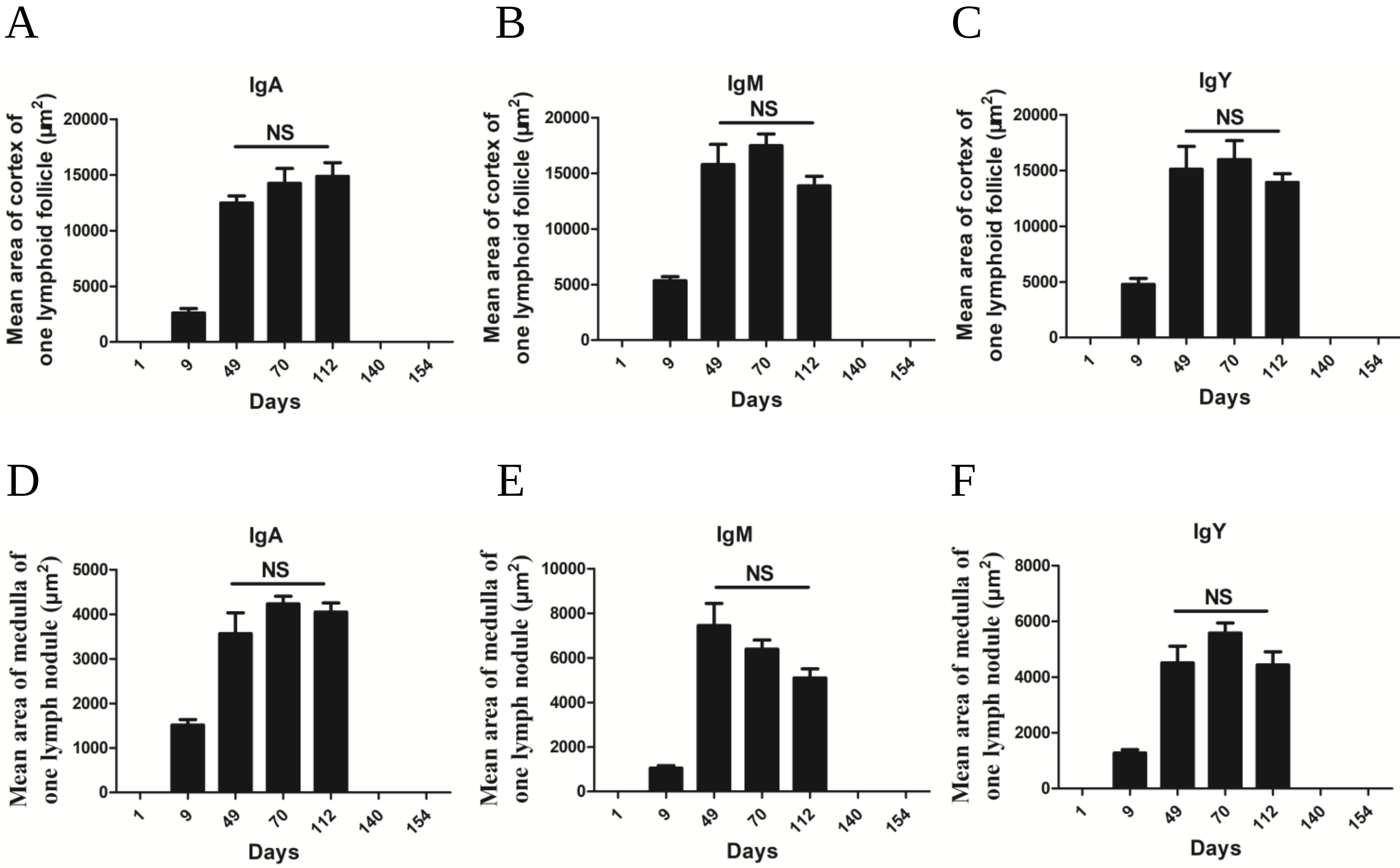

Figure S2. Age-related changes in immunostained sections of the spleen at different development stages. (A-C) IgA, IgM and IgY-positive medulla of lymph nodule on different days. (D-F) IgA, IgM and IgY-positive cortex of one lymphoid follicel on different days. Scale bar = 100 μm. Ten random fields per specimen were analyzed with Image Pro-Plus Software V.6, and the mean intensity fluorescence per power field was recorded. Data are means ± SEM (**P* < 0.05). Scale bars = 100 μm; n = 6.

Bu-1α

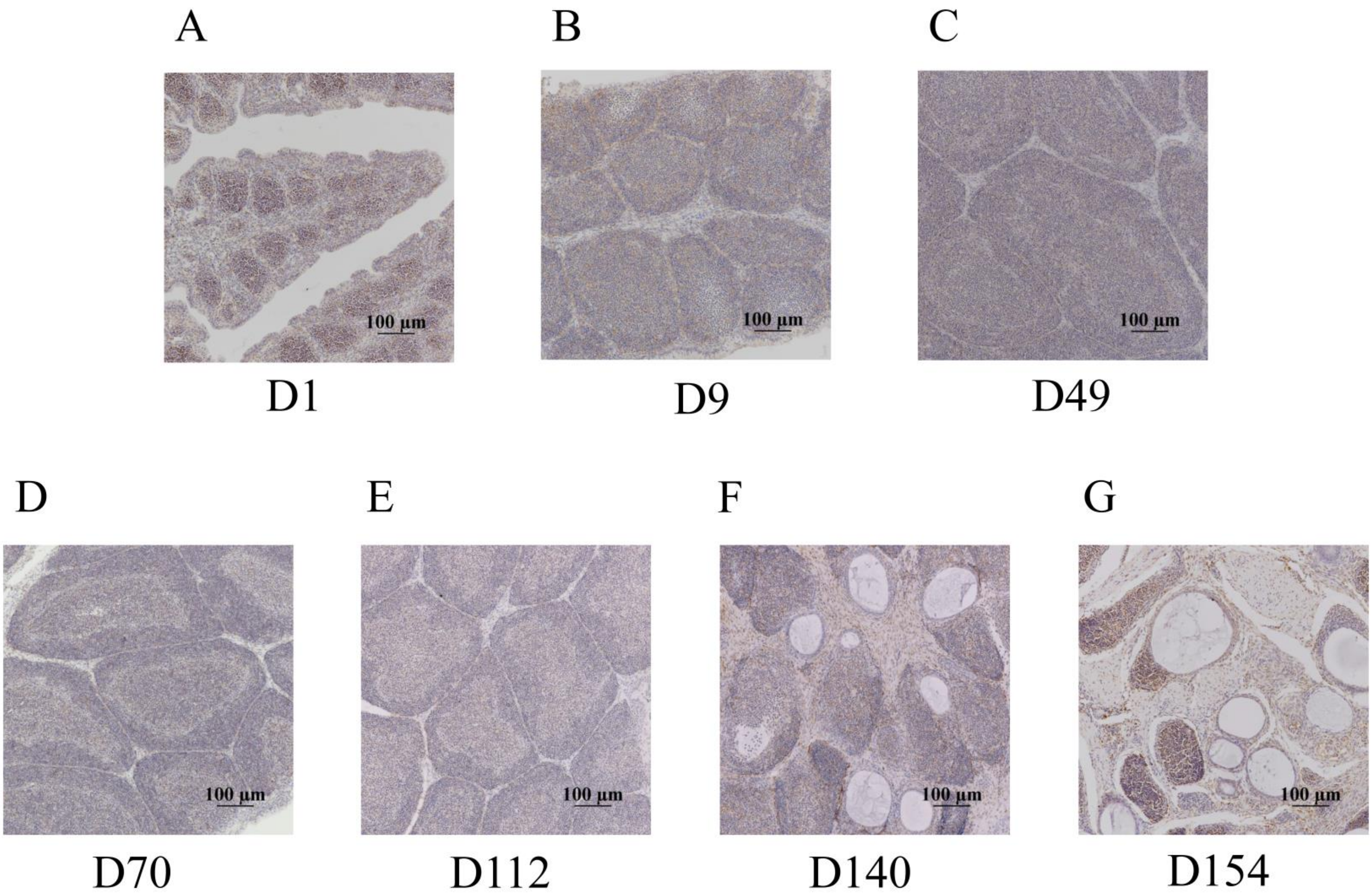

Figure S3. Age-related changes in Bu-1α-positive lymphoid follicle of the bursa of Fabricius at different development stages..

IgA

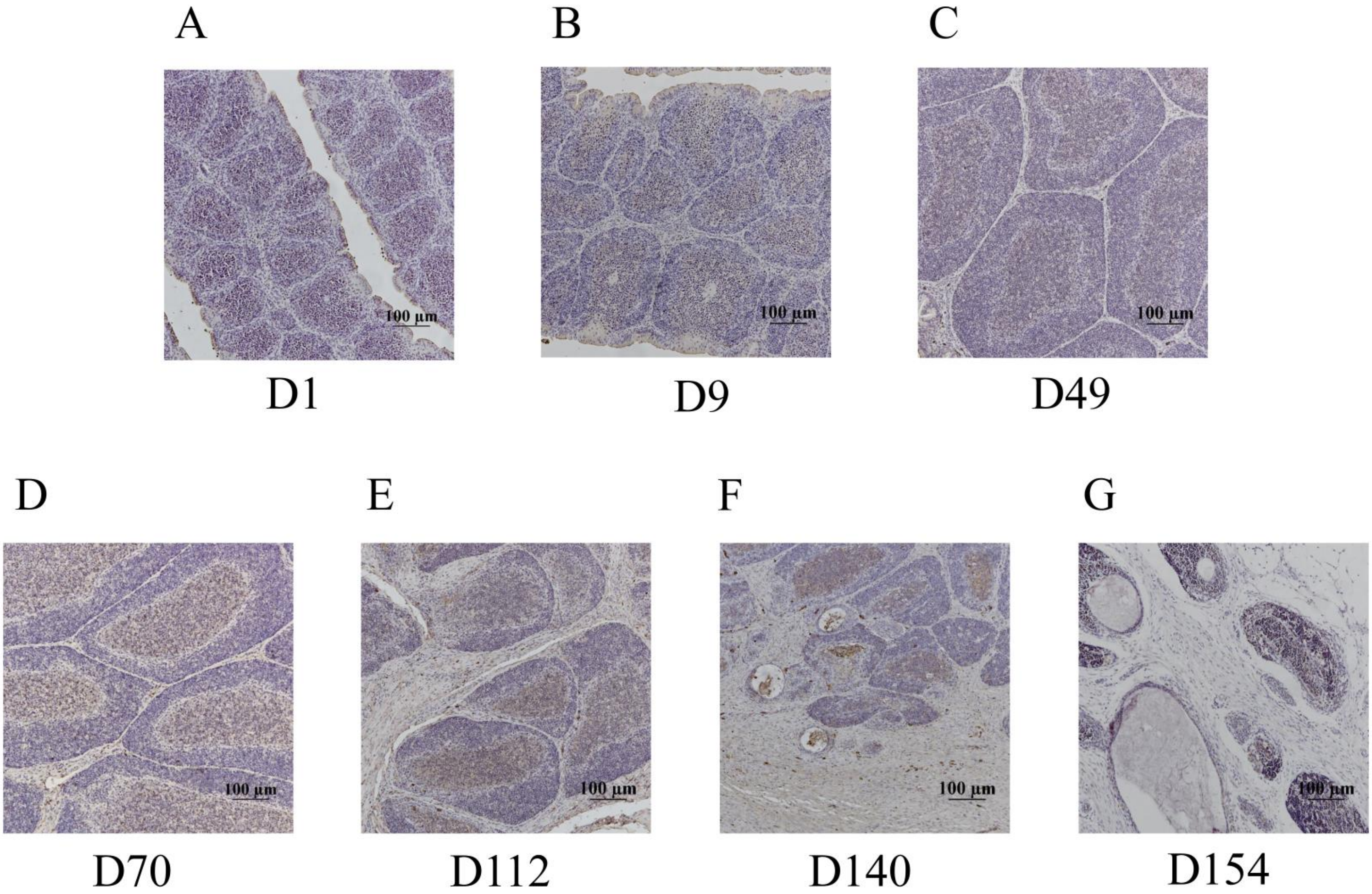

Figure S4. Age-related changes in IgA-positive lymphoid follicle of the bursa of Fabricius at different development stages.

IgM

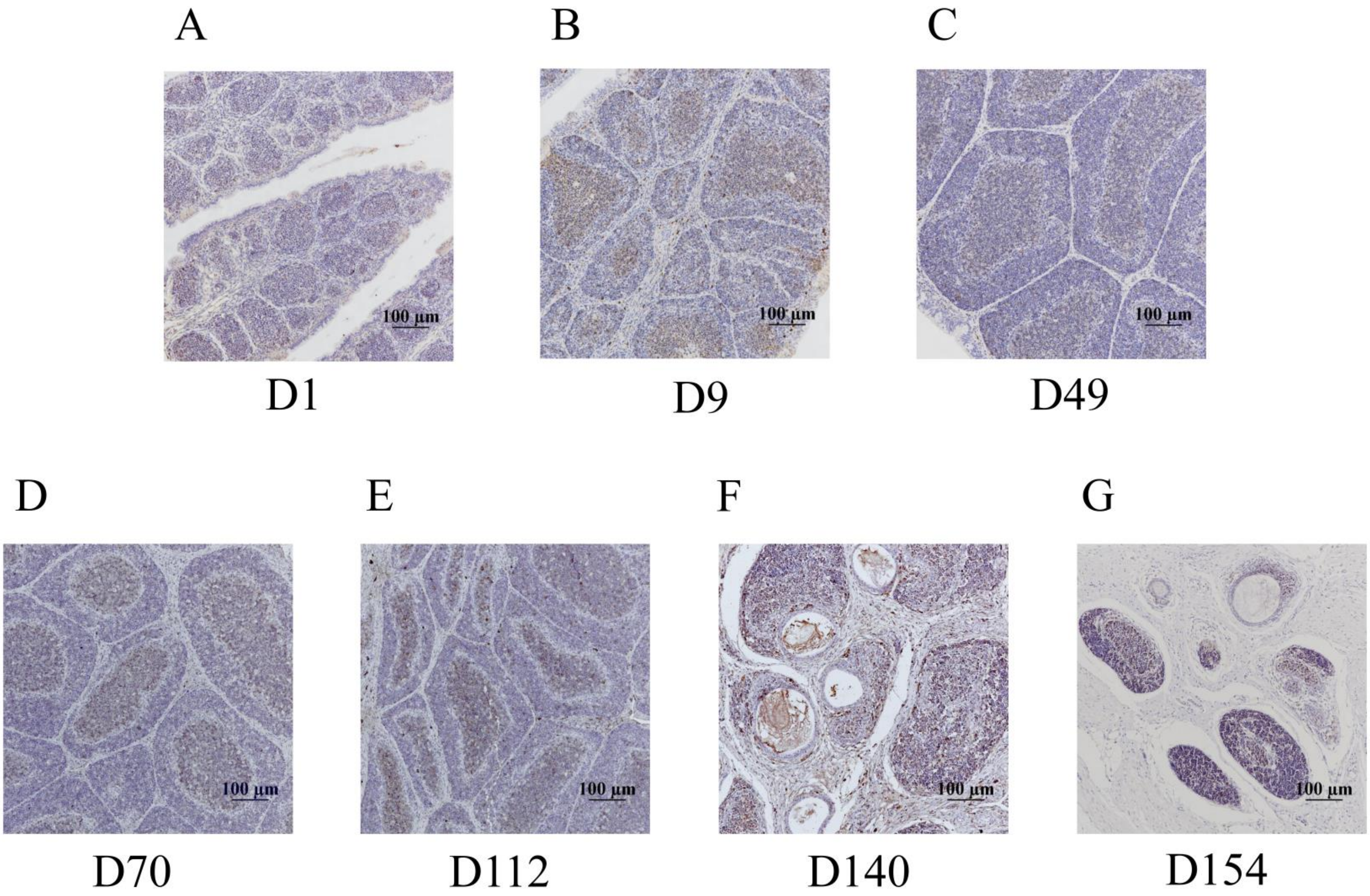

Figure S5. Age-related changes in IgM-positive lymphoid follicle of the bursa of Fabricius at different development stages.

IgY

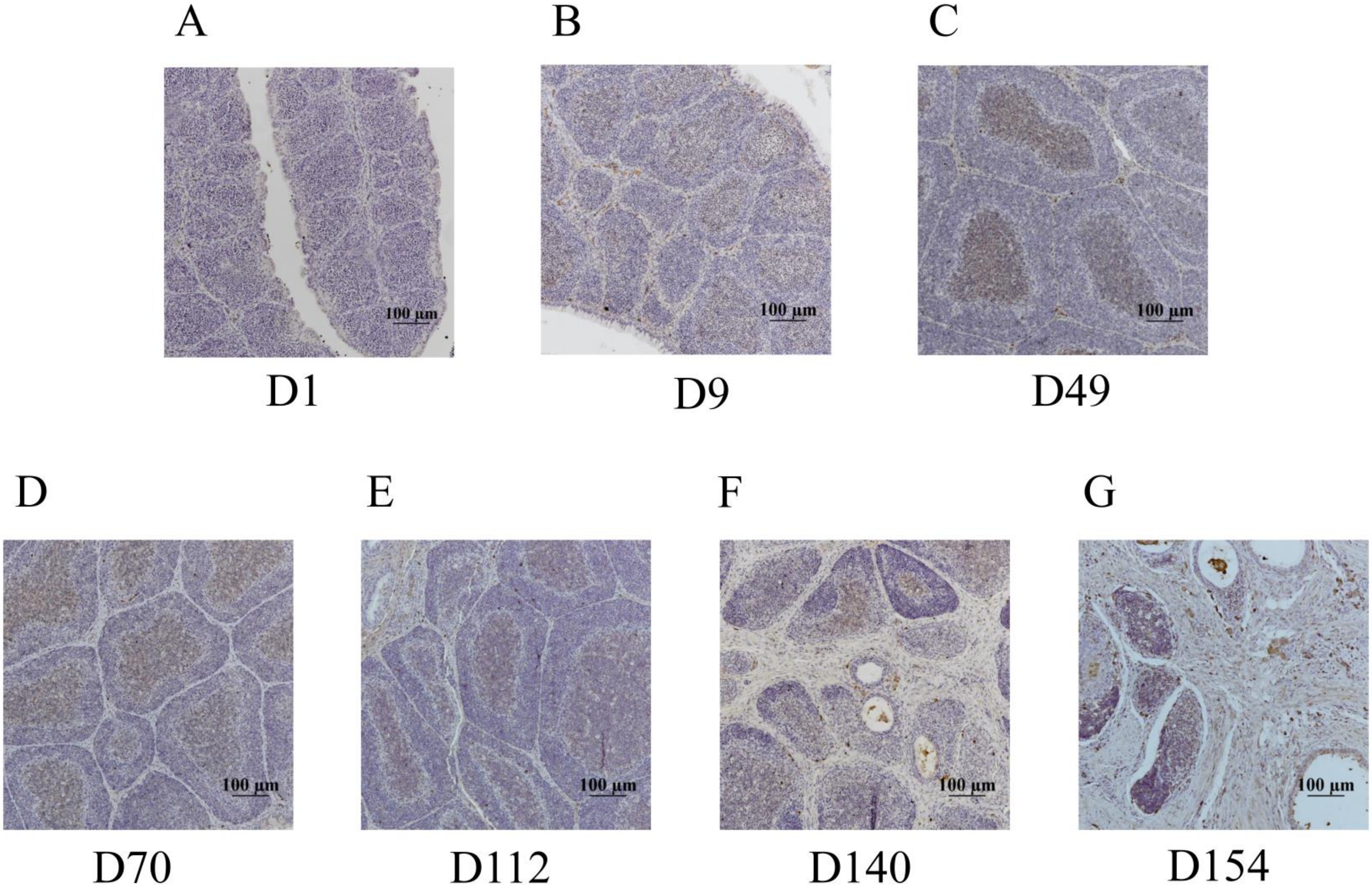

Figure S6. Age-related changes in IgY-positive lymphoid follicle of the bursa of Fabricius at different development stages.

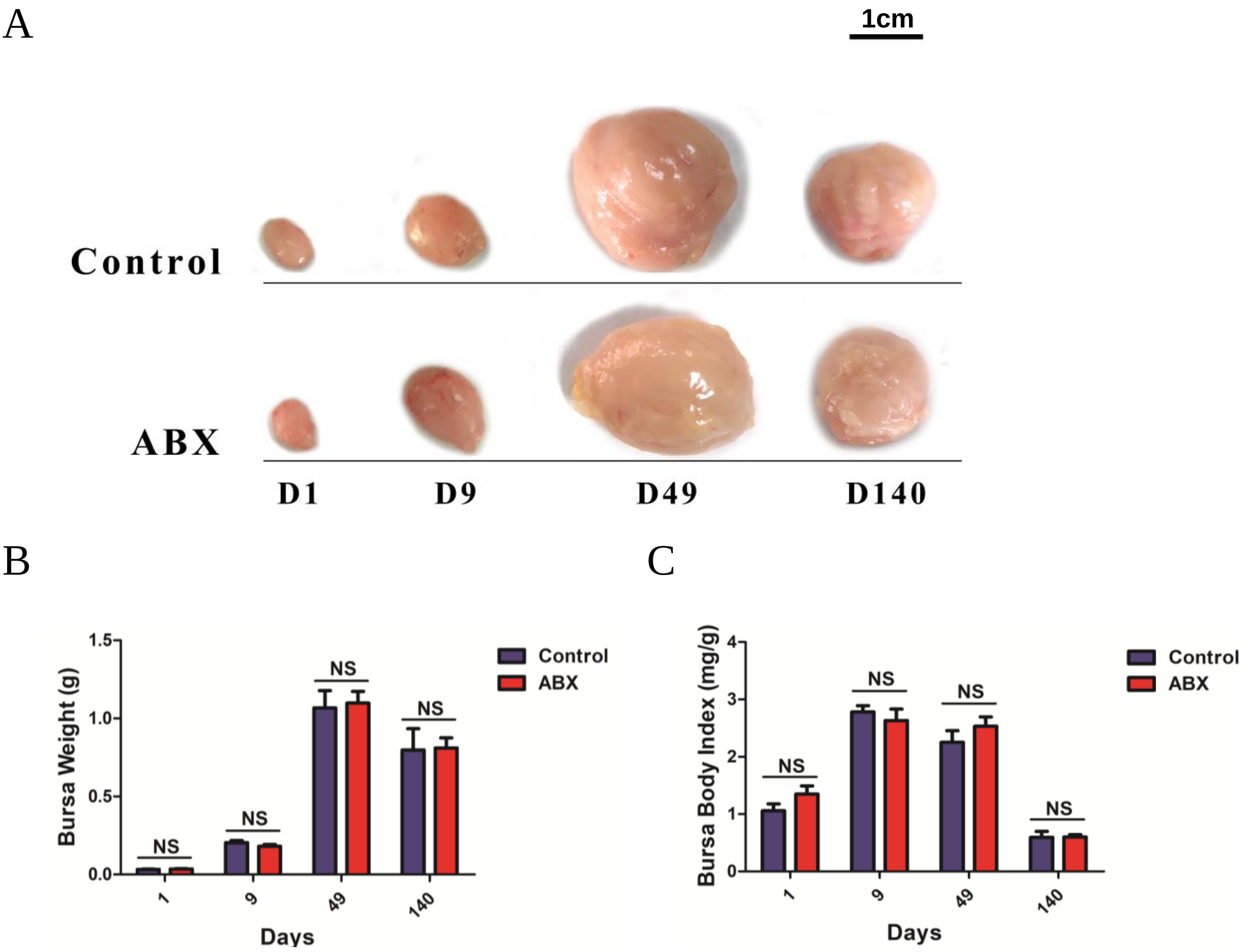

Figure S7. Changes of the size and weight of BF or the ratio of BF to body weight at different development stages under antibiotic treatment.

A

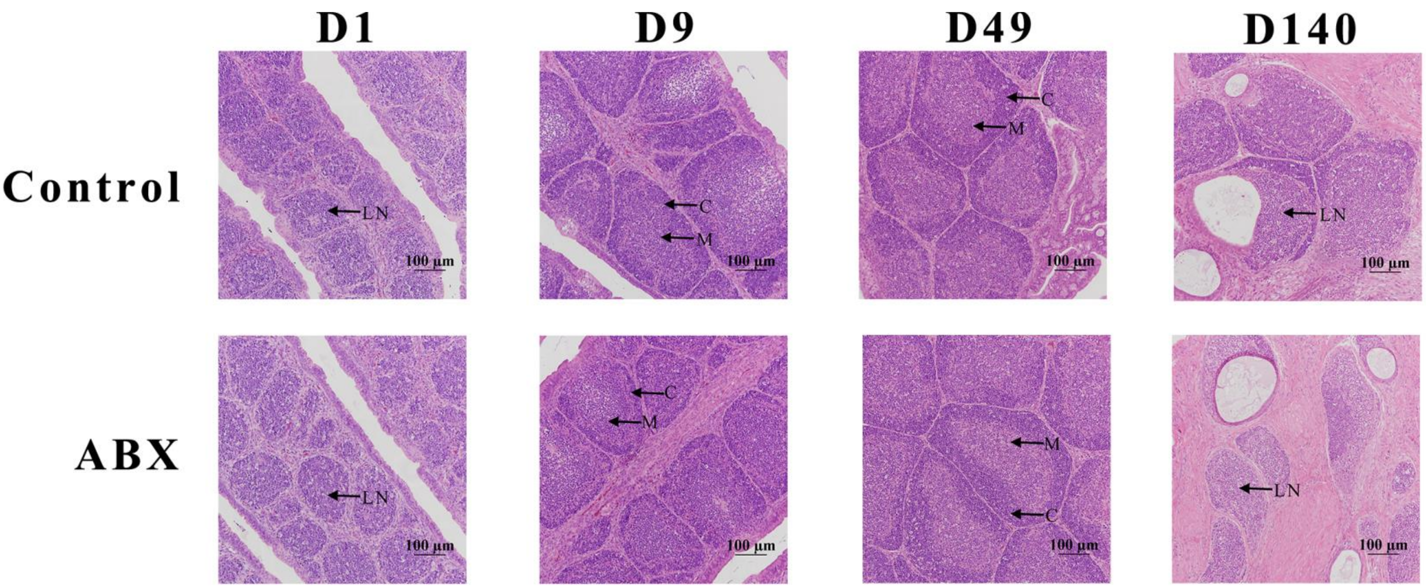

B

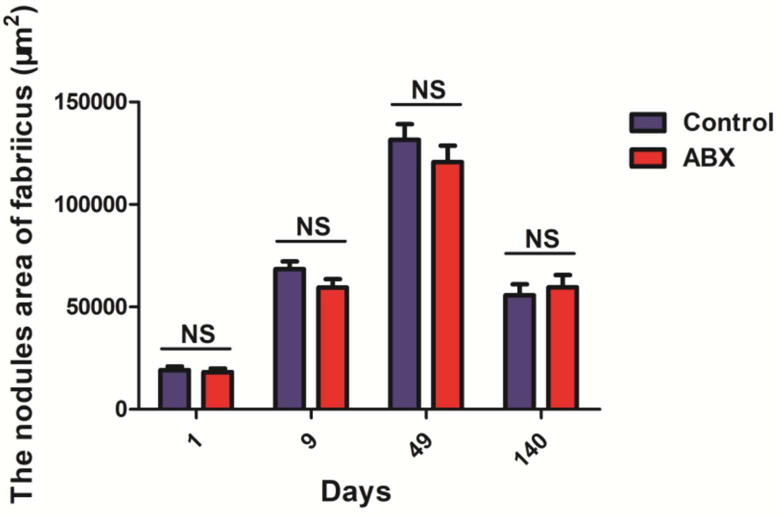

Figure S8. Changes of the nodule area of the bursa at different development stages under antibiotic treatment.

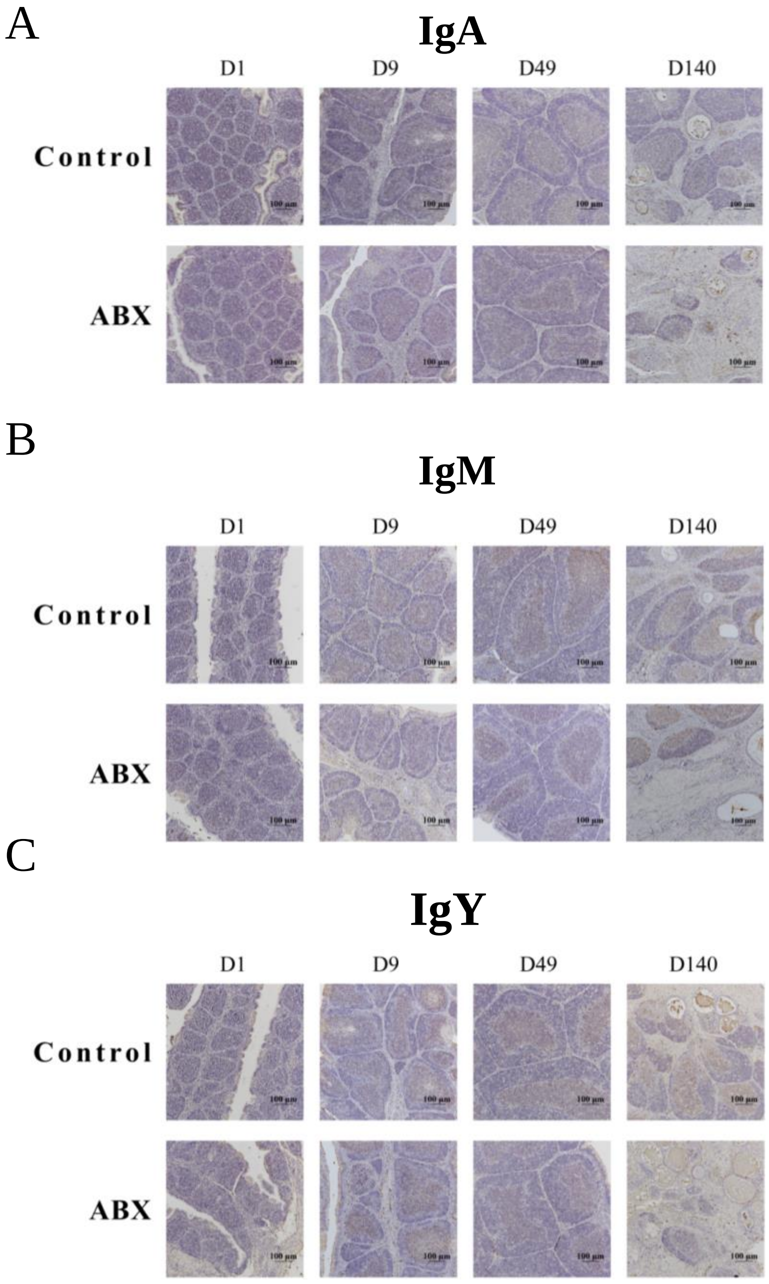

Figure S9. Changes of IgA, IgM and IgY in bursa cells after antibiotic treatment.

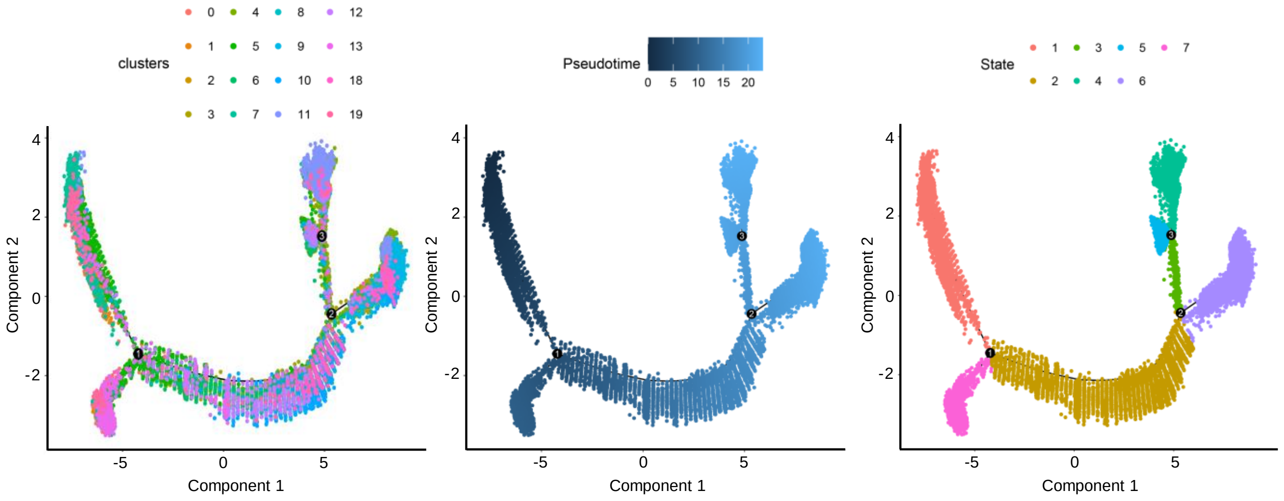

Figure S10. Changes of the developmental trajectory and differentiation gene expression pattern of large B cells, small B cells, and unnamed B cells under antibiotic treatment.

Table S1 Histological measurement of fabricius at different ages

**Age in days**	**The cortical thickness of fabricius (μm)**	**The medullary thickness of fabricius (μm)**
1	—	—
9	41±4[b]	143.82±6.64[b]
49	71±4[a]	291.45±17.14[a]
70	80±6[a]	294.4±23.39[a]
112	66±8[a]	353.78±23.64[a]
140	—	—
154	—	—

Note：n=6. Data are means ± SE. Data [a-c]Different superscripts letters represent different ages significant differences in the same column ($P< 0.05$). "—" Represents an unmeasurable scale.
